# Applications of sequencing technology in clinical microbial infection

**DOI:** 10.1111/jcmm.14624

**Published:** 2019-09-02

**Authors:** Xiaoling Yu, Wenqian Jiang, Yang Shi, Hanhui Ye, Jun Lin

**Affiliations:** ^1^ Department of Infectious Diseases Mengchao Hepatobiliary Hospital of Fujian Medical University Fuzhou China; ^2^ Institute of Apply Genomics Fuzhou University Fuzhou China; ^3^ School of Basic Medical Sciences Fujian Medical University Fuzhou China; ^4^ Fujian Key Laboratory of Marine Enzyme Engineering Fuzhou University Fuzhou China

**Keywords:** clinical microbial infection, next‐generation sequencing, third‐generation sequencing

## Abstract

Infectious diseases are a type of disease caused by pathogenic microorganisms. Although the discovery of antibiotics changed the treatment of infectious diseases and reduced the mortality of bacterial infections, resistant bacterial strains have emerged. Anti‐infective therapy based on aetiological evidence is the gold standard for clinical treatment, but the time lag and low positive culture rate of traditional methods of pathogen diagnosis leads to relative difficulty in obtaining the evidence of pathogens. Compared with traditional methods of pathogenic diagnosis, next‐generation and third‐generation sequencing technologies have many advantages in the detection of pathogenic microorganisms. In this review, we mainly introduce recent progress in research on pathogenic diagnostic technology and the applications of sequencing technology in the diagnosis of pathogenic microorganisms. This review provides new insights into the application of sequencing technology in the clinical diagnosis of microorganisms.

## 1 THE STATUS OF CLINICAL MICROBIOLOGICAL INFECTION

Infectious diseases are caused by pathogenic microorganisms, such as bacteria, viruses, parasites or fungi that can be spread between humans and animals or transmitted from animals to humans. Various pathogenic microorganisms were found most frequently from the end of the 19th century to the beginning of the 20th century.^1^, ^2^ The pioneers of microbiology, represented by Louis Pasteur, first discovered the existence of microorganisms and revealed the relationship between microbial infection and disease.

The vast majority of infectious diseases are caused by bacterial infections, which had a very high mortality rate before the discovery of antibiotics. In 1928, Alexander Fleming discovered that a secretion from *Penicillium notatum* that he named penicillin could inhibit *Staphylococcus*.^3^ Clinical trials of *Penicillium* isolates began in the 1940s. Penicillin promoted the treatment of infectious diseases and stimulated the search for other types of antibiotics.^4^ The discovery of antibiotics was a turning point in human history. Regrettably, the effects of these miraculous drugs have gradually been lost with the rapid emergence of antibiotic‐resistant strains.^5^ Shortly after the introduction of penicillin in the 1940s, penicillin‐resistant *Staphylococcus aureus* (*S aureus*) appeared; similarly, a strain of *Mycobacterium tuberculosis* resistant to streptomycin appeared shortly after the discovery of streptomycin.^6^ In addition to bacteria, viruses and fungi are also common pathogenic microorganisms in the clinic.

A statistical analysis of several severe infectious diseases, such as tuberculosis, malaria and AIDS, has been conducted by the World Health Organization (WHO). According to the WHO, in 2017, 10 million new tuberculosis cases were reported, resulting in 1.6 million deaths.^7^ There were 219 million malaria cases in 2017, and the death toll from malaria was 435 000.^8^ A total of 36.9 million people were living with HIV worldwide in 2017, resulting in 940 000 deaths.^9^ In addition, *S aureus* is one of the most common pathogens leading to human infection and the primary cause of bacteraemia, pneumonia, infective endocarditis, and skin and soft tissue‐related infections.^10^ The Centers for Disease Control and Prevention (CDC) in the USA has reported that the number of infections caused by *S aureus* is second only to the number of infections caused by *Escherichia coli*. Another common bacterium, *Pseudomonas aeruginosa* (*P aeruginosa*), a conditional pathogen, is one of the main causes of nosocomial infection; *P aeruginosa* can survive in a variety of environments, including surfaces in medical facilities, owing to its adaptability and antibiotic resistance.^11^ *Pseudomonas aeruginosa* affects more than 2 million patients, causing approximately 90 000 deaths each year.^12^

**Figure 1:**
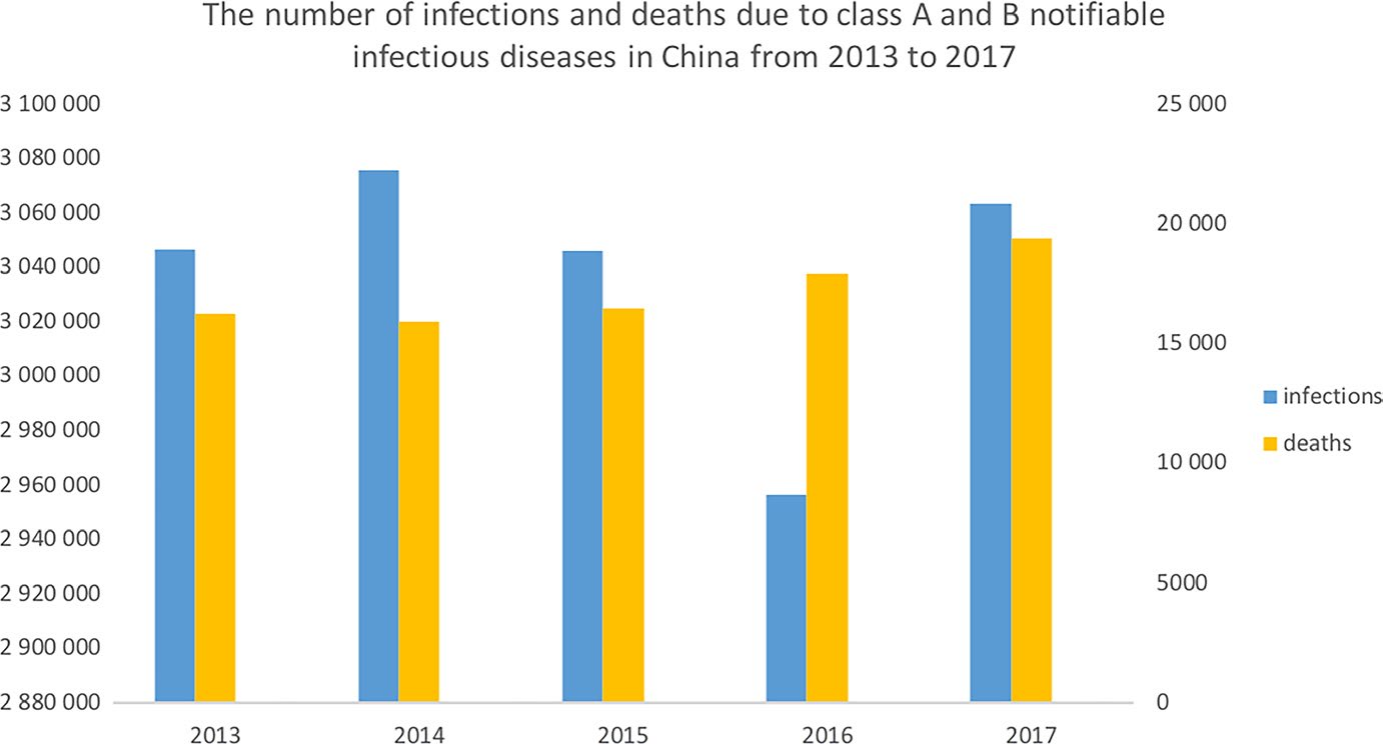
The number of infections and deaths due to class A and B notifiable infectious diseases in China from 2013 to 2017. The abscissa indicates the year, the left ordinate represents the number of infections, and the right ordinate represents the number of deaths

According to the China National Statistics Bureau, from 2013 to 2017, the number of infections and deaths due to class A and B notifiable infectious diseases in China fluctuated at approximately 3 million, but the number of deaths from these infectious diseases is increasing^13^ (Figure 1).

In recent years, with increasing numbers of drug‐resistant pathogens and a growing number of individuals abusing antibiotics in the clinic, the issue of drug resistance has become increasingly serious. The detection of pathogenic microorganisms and drug resistance has become the primary procedure for clinical anti‐infective treatment. Methicillin‐resistant *Staphylococcus aureus* (MRSA) and vancomycin‐resistant *Enterococcus* (VRE) are common and harmful antibiotic‐resistant bacteria found in the clinic. Methicillin‐resistant *Staphylococcus aureus*, which was first discovered in 1961,^14^ is resistant to multiple antibiotics and found in 74% of patients with *S aureus* infection worldwide.^15^ According to the CDC in the USA, approximately 5% of patients in hospitals in the USA carry MRSA in their nose or on their skin. Vancomycin is well known as the last line of defence against antibiotic‐resistant bacteria, and VRE‐related infections have caused a serious impact on human health as they were first reported in the 1980s.^16^ *Enterococcus* can cause a variety of clinical diseases, such as urinary tract infections, bacteraemia, endocarditis, and abdominal and pelvic infections.^17^

## 2 RESEARCH PROGRESS IN PATHOGENIC DIAGNOSTIC TECHNIQUES

Anti‐infective therapy based on aetiological evidence, including the genus, species and drug resistance of a bacterium, is the gold standard for clinical treatment. Traditional methods of pathogenic microbial detection include culture and separation, biochemical and serological detection, immunology and nucleic acid detection.^18^ Traditional aetiological diagnosis determines drug resistance by collecting clinical specimens, culturing positive pathogenic microorganisms and then conducting drug sensitive tests. Therefore, traditional aetiological diagnosis has certain defects. First, obtaining a traditional aetiological diagnosis takes a long time, and reports on bacterial detection are lagging, which results in an inability to guide anti‐infection treatment plans in sufficient time. Second, the positive detection rate with traditional aetiological diagnosis is low, and it is relatively difficult to obtain aetiological evidence. Third, in vitro culture medium is distinct from the environment and conditions of microbial growth in vivo and cannot fully reflect infection in vivo. Fourth, some infectious diseases are not caused by the presence or excessive reproduction of a single pathogenic microorganism but by dysbiosis of the flora at the lesion site, resulting in an imbalance in the population of multiple microorganisms. Furthermore, different microorganisms adapt to different media and culture conditions, and traditional in vitro culture can obtain only the dominant growing microorganisms; however, many microorganisms in nature still cannot be cultured.

In recent years, with the spread of infectious diseases worldwide, the number of suspected infections has increased, and new pathogenic diagnostic technology is rapidly developing. Mass spectrometry for rapid microbial identification,^19^ such as matrix‐assisted laser desorption/ionization time‐of‐flight mass spectrometry (MALDI‐TOF‐MS), is used to identify bacterial genera and species,^20^ but the limitation of this technique is that accurate identification depends on a well‐established database, and the organism identified with this technology must be a cultured microorganism.^21^ Molecular diagnostic techniques, such as lateral‐flow immunoassay (LFIA), combine microbial antigens with labelled antibodies,^22^ and real‐time polymerase chain reaction is used for the quantitative determination of specific microorganisms.^23^, ^24^ These detection techniques are limited to identifying the genera and species of specific pathogenic microbes.

## 3 THE DEVELOPMENT OF SEQUENCING TECHNOLOGY AND ITS CURRENT STATUS

Sequencing technology is used to determine the primary structure of biomacromolecules such as nucleic acids, proteins and polysaccharides. The most common sequencing technique is nucleic acid sequencing, including DNA and RNA sequencing, which determines the order of nucleotides in nucleic acid sequences. DNA sequencing technology has evolved from first‐generation DNA sequencing to the current fourth‐generation DNA sequencing.

Maxam‐Gilbert sequencing, Sanger dideoxy sequencing, fluorescence automated sequencing and hybrid sequencing are collectively known as first‐generation DNA sequencing technology. In the mid‐ and late 1970s, sequencing technology began to gradually mature. In 1977, Maxam and Gilbert established Maxam‐Gilbert sequencing based on chemical fracture.^25^ In the same year, Sanger proposed the dideoxy chain termination method.^26^ In 1986, Smith et al^27^ developed a semi‐automated method for DNA sequence analysis based on the principles of Sanger sequencing and fluorescence detection. With the development and improvement of fluorescence automatic sequencing technology, DNA sequencing instruments widely used for first‐generation sequencing are currently based on capillary electrophoresis and fluorescent labelling.

With completion of the Human Genome Project, the throughput of traditional sequencing methods has been unable to meet genome sequencing needs. In the mid‐ and late 1990s, next‐generation sequencing (NGS) emerged. Next‐generation sequencing has the potential to accelerate biological and biomedical research, increase throughput and reduce production scale and labour costs.^28^ It is suitable for not only genome sequencing and genome re‐sequencing but also transcriptome analysis (RNA‐Seq), the characterization of DNA‐protein interaction (ChIP‐sequencing) and epigenomics^29^ .

**Figure 2:**
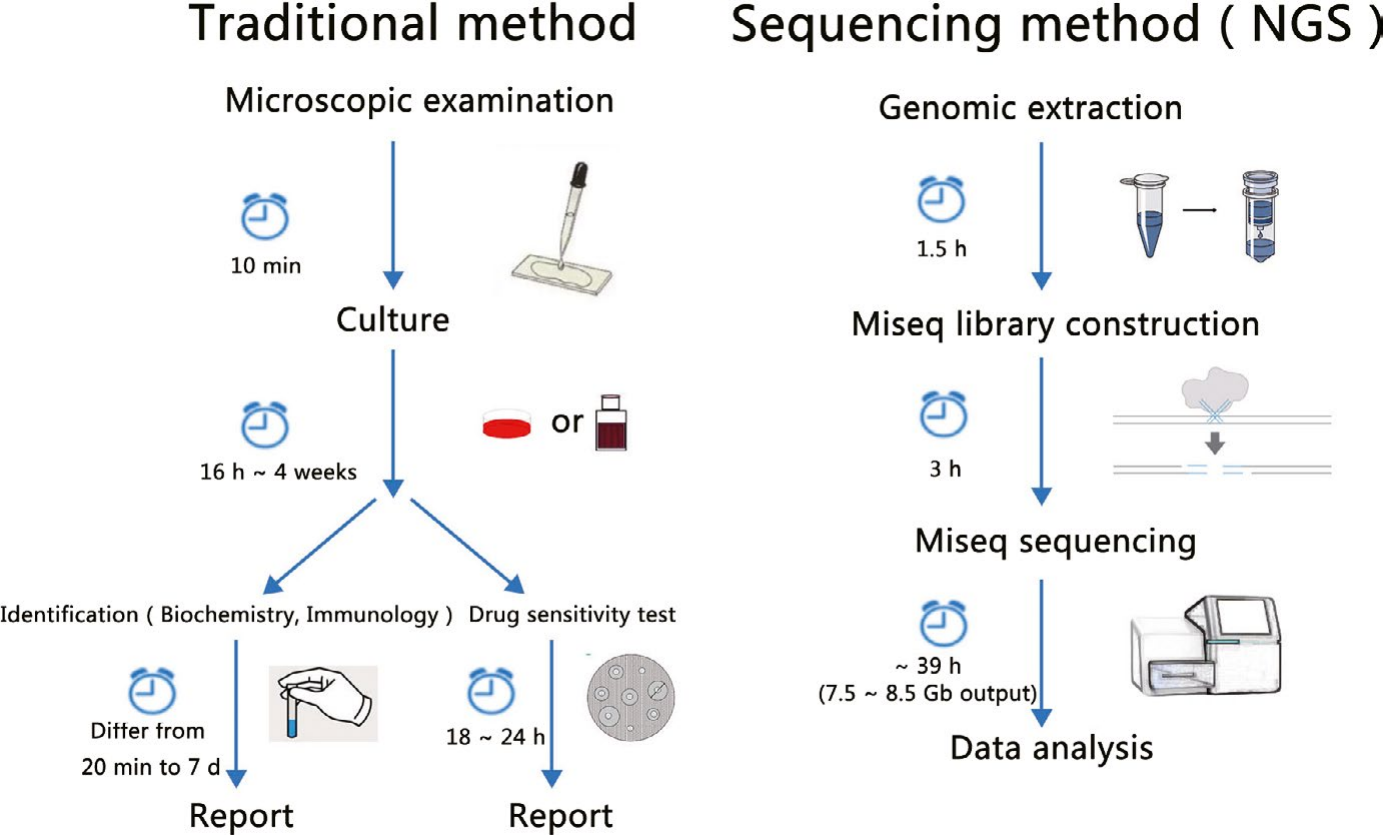
Comparison of the traditional method and next‐generation sequencing (NGS)

From 2008 to 2009, sequencing technology using different methods from those of the second‐generation platform was first described as ‘third‐generation’ sequencing.^30^ Unlike second‐generation sequencing, which requires long‐chain DNA, third‐generation sequencing machines, such as the HeliScope,^31^ Nanopore^32^ and PacBio^33^ systems, read nucleotide sequences at the single‐molecule level. Third‐generation sequencing can produce longer reads than current sequencing technology,^34^ and its portability and sequencing speed are also important advantages.^35^ At present, third‐generation sequencing technology still has some limitations, which include its reliance on the activity of high‐cost DNA polymerase and an error rate that is much higher than that observed in NGS.^36^

## 4 APPLICATION STATUS OF SEQUENCING TECHNOLOGY IN PATHOGENIC DIAGNOSIS

### 4.1 First‐generation DNA sequencing

Sanger sequencing was the main sequencing technique used between 1975 and 2005 and the gold standard for all sequencing technologies. Ribosomal RNA, especially 16S rRNA and 23S rRNA, is the most useful phylogenetic marker for pathogenic diagnosis by Sanger sequencing.^37^ The most commonly used molecule for microbial species identification is 16S rRNA, which is extremely abundant in bacteria. The 16S rRNA sequence is approximately 1.5 kb in length^38^ and highly conserved in structure and function with multiple variable regions.^39^ Therefore, the genera and species of different pathogenic bacteria can be identified by 16S rRNA sequencing. Currently, 16S rRNA gene analysis of bacteria is primarily achieved by sequencing the variable regions of the 16S rRNA gene.^40^ Single clones from microorganisms obtained by culture can be identified by Sanger sequencing after amplification with 16S universal primers. The full‐length 16S rRNA gene in bacteria can be sequenced by Sanger sequencing, but the drawback of this strategy is that the bacterial culture (BC) must be pure; otherwise, the bacterium cannot be identified. The positive rate of clinical culture is very low, most microorganisms cannot be detected, and a mixture of 16S genes cannot be used to distinguish species. This time‐consuming strategy of cultivation and sequencing cannot meet the timeliness required of clinical tests. All of the above defects seriously affect the scope of the application of first‐generation DNA sequencing.

### 4.2 Next‐generation metagenomic DNA sequencing (mNGS)

Based on the popularity and maturity of NGS, professional companies are already providing clinical microbiological testing based on mNGS. Next‐generation sequencing technology, which is represented by the Illumina system, is sequencing by synthesis that captures the base‐linked fluorescent signal on a cluster of PCR molecules and converts it into base information (Figure.2) .^41^

Because of the limitation of culture conditions and the influence of pre‐antibiotic treatment, the positive rate of clinical microbial testing is low, and it is impossible to detect rare, unknown and new pathogens. Aetiological diagnosis by mNGS is carried out to determine the genera of microorganisms by sequencing the microbial DNA of various specimens in clinical samples.^42^ As NGS does not depend on cultured microorganisms, some dead and uncultured microorganisms could also be detected; on the other hand, high‐throughput detection greatly reduces the cost and time of microorganism identification, and the accuracy of NGS is better than that of ELISA, PCR and hybridization chips. In 2010, whole‐genome sequencing (WGS) of bacterial pathogens began to migrate from research laboratories to public health practice.^43^ One of the earliest applications of WGS in public health was the analysis of the epidemiology of hospital‐acquired infections, such as *Acinetobacter baumannii* infection, which broke out in a British hospital in 2010.^44^ In May 2016, the Federal Drug Administration (FDA) issued the ‘Infectious Disease Next Generation Sequencing Based Diagnostic Devices: Microbial Identification and Detection of Antimicrobial Resistance and Virulence Markers (draft)’ guideline.^45^

In recent years, mNGS has been applied in clinical practice. The world's first case of pathogen diagnosis using NGS occurred in 2013. A 14‐year‐old boy with severe combined immunodeficiency (SCID) was hospitalized for fever and headache several times over 4 months. His doctors could not identify the cause of his disease by diagnostic examination, including brain biopsy. Finally, a *Leptospira* species that could cause encephalitis was discovered by NGS, after which doctors cured the boy with a large dose of penicillin.^46^ Food‐borne pathogens are the major cause of morbidity and mortality worldwide. Next‐generation sequencing can be used for real‐time sequencing during pathogenic microbial outbreaks.^47^ For example, in a food poisoning incident in Nanjing, China, *Salmonella Schwarzengrund* was isolated from diarrhoeal patients, and NGS was used to confirm the cause of the outbreak and trace contamination.^48^ The incidence of sepsis, a seriously life‐threatening infection with a high fatality rate, has been increasing recently.^49^ It is estimated that more than 30 million people worldwide are affected by sepsis each year, possibly leading to 6 million deaths.^50^ Gosiewski et al used NGS to analyse blood samples from 23 healthy volunteers and 62 patients with sepsis and found significant differences in bacterial diversity between the sepsis patients and healthy volunteers. Among the healthy volunteers, anaerobic bacteria accounted for the main proportion (76.2%) of bacterial species, and the abundance of *Actinobacteria phyla* was significantly higher than that in patients with sepsis (76.3% in healthy volunteers and 31.0% in the sepsis patients), while among the sepsis patients, most species (75.1%) were aerobic or microaerobic microorganisms, and the abundance of phyla in the *Proteobacteria* was significantly higher in patients with sepsis than in the healthy volunteers (16.4% in healthy volunteers and 60.1% in sepsis patients).^51^ Grumaz et al^52^ conducted a study based on sepsis in intensive care units (ICUs) to identify infectious microbes from seven sepsis patients by the unbiased sequence analysis of free circulating DNA from plasma through NGS. This method is expected to serve as a diagnostic platform for critically ill patients with blood infections. Long et al used NGS technology to identify pathogens in plasma samples from 78 ICU patients. The overall diagnostic sensitivity increased significantly from 12.82% (10/78) in BC alone to 30.77% (24/78) with NGS alone, which provided more useful information to establish patient treatment plans.^53^ Pulmonary disease is a common and widespread disease in daily life. Lung transplant patients are a vulnerable group of immunosuppressed patients susceptible to frequent respiratory infections. The viral aetiology of infection in four lung transplant patients (three Rhinovirus A infections and one Rhinovirus B infection) was determined with mNGS,^54^ emphasizing its potential for viral diagnosis in infections of previously unknown aetiology and complex diagnostic situations. *Klebsiella pneumoniae* (*K pneumoniae*) is the leading cause of pneumonia in hospitalized patients, but the bacterial factors required to cause disease from *K pneumoniae* are poorly understood. Bachman et al^55^ used insertion site sequencing to combine transposon mutagenesis with high‐throughput sequencing; the results indicated that the regulation of outer membrane components and the synthesis of essential amino acids in the *K pneumoniae* host are critical for its fitness in the lung, which is important for the development of new antibiotics against *K pneumoniae*. A skin ulcer is a surface disease characterized by the long‐term non‐healing of skin, liquefaction infection and necrosis of skin tissue defects. The risk of foot infection in diabetic patients is much higher than that in normal people. Malone et al^56^ used NGS to analyse the microbiome of infected diabetic foot ulcers (DFUs), which confirmed that short‐term DFUs have a simpler microbiome composed of pyogenic cocci, but chronic DFUs have a highly polymicrobial microbiome and that the duration of the DFU can be used as a guide for antibacterial therapy. In addition to the above cases, mNGS has been applied to other clinical infections, but there are still some defects and technical problems with this technique.

The specific technical problems in the diagnosis of pathogens by mNGS include the following. First, mNGS is limited to roughly judging the species of pathogenic microorganisms and estimating the approximate proportion of those microorganisms, and the results of different tests or from different labs may vary. Second, mNGS is unable to directly detect RNA viruses, because the RNA needs to be reverse transcribed to cDNA.^57^ Reverse transcription is necessary to sequence RNA viruses, but this process will take extra time. At the same time, the reads from mNGS are relatively short, so it is difficult to obtain information such as the full‐length antibiotic resistance gene sequences in pathogenic microorganisms, and it is impossible to associate the drug resistance gene with the corresponding pathogenic microorganism species. Moreover, short‐read sequencing will introduce bias.^58^, ^59^ Third, the detection rate of some low‐content intracellular bacteria, such as *M tuberculosis*, *Legionella*, *Brucella* and fungi with thick cell walls, is low. Fourth, mNGS requires multiple rounds of PCR amplification in the process of library construction, so cross‐contamination issues are prone to occur. Fifth, a certain proportion of the extracted macrogenomic DNA is derived from dead bacteria, and mNGS cannot determine whether the detected sequence is from living or dead bacteria. Sixth, because of the high‐throughput nature of mNGS, a sufficient number of samples are needed to run the sequencing machine, which means that sequencing with the machine cannot start at any time. Furthermore, the sequencing time is slightly longer than that of other techniques; for example, to sequence using the Illumina system in 300 bp paired‐end mode, the time from booting the sequencing system to data acquisition is more than 60 hours, while for infected patients, the need to obtain aetiological evidence is urgent. Therefore, the diagnostic results of mNGS have limited clinical reference value.

### 4.3 Long‐read third‐generation sequencing technology

The 16S rRNA gene sequence commonly used for microbial species identification is 1.5 kb in length^60^; however, the longest read length of mNGS systems, such as Illumina and BGI sequencers, is only 300 bp. The reads from mNGS are short, and computer analysis based on short sequences cannot completely determine the real sequence. Therefore, both machines can identify microorganisms to only the genus level and not to the species level from 16S rRNA. Metagenomic sequencing uses short reads to search the database for species identification, which is prone to error mapping.^61^ Long‐read third‐generation sequencing technology can measure reads longer than 1 Mb, which can be used not only for microbial metagenome sequencing but also to directly measure full‐length 16S rRNA and identify pathogenic bacteria to the species level.

Third‐generation sequencing technology represented by the Nanopore system was launched in 2015. Compared with NGS, third‐generation sequencing produces longer reads, and the results can be obtained in a shorter time. The Nanopore system is an electrical signal‐based sequencing technology.^62^ Protein‐based nanopores (microscopic pores, which essentially form channels on the membrane) are embedded in a synthetic membrane and immersed in electrophysiological solution to allow ion currents to pass through the nanopore. The current is interfered when DNA or RNA molecules pass through it, causing a characteristic change in the current signal. In this process, the signal is analysed in real time to determine the base sequence of the DNA or RNA strand that is passing through the pore, which allows the entire DNA or RNA sequence to be determined.^63^, ^64^

Nanopore sequencing technology effectively addresses the defects of mNGS in the field of aetiological diagnosis, directly sequencing reads more than 1 Mb in length,^65^ which allows the identification of species of pathogenic microorganisms by 16S rRNA sequencing. Nanopore sequencing technology eliminates the effort and time needed for reverse transcription because the reverse transcription of RNA into cDNA is not required; the Nanopore system can perform RNA sequencing directly.^66^ The throughput is very high, and the latest version of the Nanopore RNA direct sequencing chip can obtain 1 million full‐length RNA sequences at a time. Sequencing results are obtained at a fast speed and available in a few hours. Furthermore, Nanopore sequencing can be started at any time, and the MinION sequencer is a miniature palm sequencer with a single chip flux that fits 1‐2 specimens.^67^

Pacific Biosciences (PacBio) is another long‐read sequencing technology system that uses a single‐molecule, real‐time (SMRT) chip as a carrier for sequencing by synthesis. Single‐molecule, real‐time sequencing begins with preparation of the SMRTbell template library. The SMRTbell template is a closed, single‐stranded, circular DNA with hairpin adapters at both ends; the SMRTbell diffuses into a sequencing unit called a zero‐mode waveguide (ZMW) when it is loaded into the SMRT cell, and single polymerases that bind to the template are anchored at the bottom of each ZMW. Four different fluorescently labelled dNTPs subsequently randomly enter the bottom of the ZMW, and after the polymerase incorporates the labelled nucleotide and cleaves its fluorophore, light pulses corresponding to the doped bases are generated in the thin region, and each pulse has its own colour intensity and duration to enable identification of the base.^68^, ^69^ The cost of PacBio sequencing is very high, and there have been only a few reports of microbial metagenomics studies using PacBio sequencing.^64^ However, taking 16S rRNA sequencing as an example, only 5000 circular consensus sequencing (CCS) reads are obtained per sample during sequencing.^70^ For the same cost, the throughput screening efficiency of Nanopore direct RNA sequencing can be much higher.

The PacBio system is rarely used for infection diagnosis because of the very large size of the machine, the expensive hardware, cumbersome library construction and its inability to directly detect RNA. At present, the Nanopore system is widely used.

Due to the above advantages, Nanopore sequencing technology has been widely used in the field of epidemic outbreak investigation over the past 2 years to detect infectious pathogens, antimicrobial drug resistance and other infectious areas of concern. For the rapid and real‐time monitoring of outbreaks, researchers conducted real‐time dynamic genomic monitoring of the Lassa fever epidemic in Nigeria^71^ and the Guinea Ebola virus epidemic^72^ through a small portable Oxford Nanopore MinION device. Through the use of direct RNA sequencing, a team of microbiologist led by John Barnes of the US CDC sequenced the complete RNA genome of the influenza A virus using Nanopore.^73^ The Prazsk I team of the Faculty of Medicine at the University of Szeged in Hungary analysed the lytic transcriptome of Varicella‐zoster virus (VZV) using the Nanopore sequencing platform, revealing the complex transcriptome structure of VZV.^74^ Moon et al^75^ used a Nanopore MinION sequencer for 16S rRNA amplicon sequencing to diagnose the first case of *Campylobacter fetus* meningitis in Korea. *Haemophilus influenza* was identified in patients with community‐acquired pneumonia by deep sequencing of the 16S rRNA gene from sputum.^76^ To determine drug resistance, Professor Arnold Bainomugisa of the University of Queensland in Australia used Nanopore sequencing technology to carry out rapid WGS of *M tuberculosis* to the genus and species level.^77^ A team led by Justin O'Grady in the UK used Nanopore sequencing technology to rapidly identify bacterial genera and species and bacterial resistance genes in patients with lower respiratory tract infections.^78^ Runtuwene et al^79^ used Nanopore sequencing to genotype the malaria parasite—*Plasmodium falciparum*—and infer its drug resistance status. The clinical development and application of Nanopore sequencing technology have become a new milestone in precise pathogen detection.

The time lag and low positive culture rate of traditional pathogen diagnosis leads to relative difficulty in obtaining evidence of pathogens. Metagenomic DNA sequencing is limited to roughly judging the microbial species and estimating its approximate proportion, and it is difficult to obtain information such as the full‐length sequence of pathogenic microbial resistance genes. The diagnostic results of mNGS have limited reference value in clinical practice. Third‐generation sequencing technology has many advantages that address the defects in NGS and traditional pathogenic diagnostic methods. Therefore, for the rapid diagnosis of clinical infectious microorganisms, the use of ‘culture‐independent’ technology combined with third‐generation sequencing must be a major developmental direction for clinical pathogenic microorganism diagnosis.

### 4.4 Outlook

In recent years, new changes in infectious diseases have taken place worldwide, and the increasing number of suspected infections and rapid increase in the rate of transmission have brought severe challenges to the diagnosis and treatment of infectious diseases. For the diagnosis of microbial infection, compared with traditional pathogenic diagnosis methods, NGS and third‐generation sequencing have the advantage of being faster, more accurate and high‐throughput and play an increasingly important role in the rapid detection and diagnosis of infectious diseases. With the continuous development of sequencing technology and the continuous improvement of the pathogenic microorganism database, infectious disease detection and epidemic investigation are expected to occur in real time, allowing for the rapid and accurate diagnosis and treatment of new, sudden and critical patients with severe infections.

## CONFLICT OF INTEREST

The authors have declared that no competing interest exists.

## AUTHOR CONTRIBUTION

WJ performed the literature research and drafted the manuscript; YS made the figure; XY and JL revised the manuscript and directed the review to be more focused; and HY and JL gave the final approval for the article to be published.
